# Solvent Properties of Water in Aqueous Solutions of Elastin-Like Polypeptide

**DOI:** 10.3390/ijms160613528

**Published:** 2015-06-12

**Authors:** Luisa A. Ferreira, James T. Cole, Christian Reichardt, Nolan B. Holland, Vladimir N. Uversky, Boris Y. Zaslavsky

**Affiliations:** 1 Analiza, Inc., Cleveland, OH 44114, USA; E-Mail: Luisa.ferreira@cleveland-diagnostics.com; 2 Department of Chemical and Biomedical Engineering, Cleveland State University, Cleveland, OH 44115, USA; E-Mails: jamejs@gmail.com (J.T.C.); n.holland1@csuohio.edu (N.B.H.); 3 Department of Chemistry, Philipps University, Marburg D-35032, Germany; E-Mail: reichardt@chemie.uni-marburg.de; 4 Department of Molecular Medicine and USF Health Byrd Alzheimer’s Research Institute, Morsani College of Medicine, University of South Florida, Tampa, FL 33612, USA; 5 Institute for Biological Instrumentation, Russian Academy of Sciences, Pushchino, Moscow Region 142292, Russia; 6 Department of Biology, Faculty of Science, King Abdulaziz University, Jeddah 21589, Saudi Arabia; 7 Laboratory of Structural Dynamics, Stability and Folding of Proteins, Institute of Cytology, Russian Academy of Sciences, St. Petersburg 194064, Russia

**Keywords:** elastin-like polypeptide, phase-transition temperature, solvent properties, solvent dipolarity/polarizability, hydrogen-bond donor acidity, and hydrogen-bond acceptor basicity, osmolyte

## Abstract

The phase-transition temperatures of an elastin-like polypeptide (ELP) with the (GVGVP)_40_ sequence and solvent dipolarity/polarizability, hydrogen-bond donor acidity, and hydrogen-bond acceptor basicity in its aqueous solutions were quantified in the absence and presence of different salts (Na_2_SO_4_, NaCl, NaClO_4_, and NaSCN) and various osmolytes (sucrose, sorbitol, trehalose, and trimethylamine *N*-oxide (TMAO)). All osmolytes decreased the ELP phase-transition temperature, whereas NaCl and Na_2_SO_4_ decreased, and NaSCN and NaClO_4_ increased it. The determined phase-transition temperatures may be described as a linear combination of the solvent’s dipolarity/polarizability and hydrogen-bond donor acidity. The linear relationship established for the phase-transition temperature in the presence of salts differs quantitatively from that in the presence of osmolytes, in agreement with different (direct and indirect) mechanisms of the influence of salts and osmolytes on the ELP phase-transition temperature.

## 1. Introduction

It is well known that interactions of any solute, ranging from small organic compounds to macromolecules, with an aqueous environment are fundamentally important for their functions *in vivo* [1,2,3]. However, our current understanding of the mechanisms underlying interactions between the aqueous solvent and solute is very limited. The solute–solvent interactions for any given solute are governed by the properties of solvent, though the contributions of different solvent properties would depend on the solute structure and physicochemical features. According to Cabot and Hunter [4], most quantitative approaches to the study of solvation phenomena have focused on the use of specially designed spectroscopic probes sensitive to changes in their environment [5,6,7]. The most widely used term for solvent classification is polarity. This is a very poorly defined term, which, according to the current definition, is the sum of all possible specific and non-specific interactions between the solvent and any potential solute, excluding interactions leading to chemical transformations of the solute [5,8]. The solute–solvent interactions include multiple types of interactions, such as electrostatic, dipole–dipole, dipole-induced dipole, hydrogen bonding and electron pair donor–acceptor interactions. It is especially important that polarity describes the potential behavior of the solvent in a relationship with the solute, which is not an absolute property of the pure solvent [8]. There is a large number of different polarity scales based on different probes and spectroscopic techniques (NMR, IR, UV/Visible absorption and emission spectroscopy, *etc.*) [4]. According to Ab Rani *et al.* [8], there is no single measure of polarity; all the polarity scales are estimates and different scales provide different estimates for the same solvent. There is no useful concept of “right” or “wrong” when comparing these scales. The test of an empirical polarity scale is its usefulness in explaining and/or predicting other solvent dependent phenomena [8].

Any single-parameter polarity scale cannot represent the multitude of possible solute–solvent interactions. Therefore Kamlet and Taft developed multi-parameter polarity scales based on Linear Solvation Energy Relationship (LSER) including three scales, such as hydrogen bond donor acidity (α) [9], hydrogen bond acceptor basicity (β) [10], and dipolarity/polarizability (π*) [11]. Combination of these three scales describes the ability of a given solvent to participate in solute–solvent interactions, *i.e.*, solvent polarity, much better than any single-parameter polarity scale. The LSER model used by Kamlet, Taft, and their co-workers may be described as:

(XYZ) = (XYZ)_o_ + *s* π* + *a* α + *b* β (1)

where (XYZ) is the solute property (solubility, reaction rate, equilibrium constant, the logarithm of a gas/solvent or solvent/solvent partition coefficient, *etc.*) in a given solvent; (XYZ)_o_ is the same solute property in a reference state; *s*, *a*, and *b* are the solute-dependent coefficients characterizing the respective influence of the π*, α, and β terms on the (XYZ) property under study. Although there are multiple examples of the successful use of Kamlet–Taft approach, the discussion of these examples is beyond the scope of this study.

It has been recently shown that typical crowding agents are capable of changing the solvent properties of aqueous media in their solutions [12]. The solvent properties of aqueous media were studied by the solvatochromic comparison approach developed by Taft, Kamlet, and others [9,10,11]. This approach is based on using a set of solvatochromic dyes with the wavelength positions of their UV/Vis absorption maxima shifting in accordance with the different solvent properties. This approach was used to quantify the solvent’s dipolarity/polarizability, hydrogen-bond donor (HBD) acidity, and hydrogen-bond acceptor (HBA) basicity in aqueous solutions of polyethylene glycols (PEGs) of different molecular mass [13], dextran, Ficoll, and other crowding agents [12]. It has been shown that the influence of crowding agents on stability, refolding, and aggregation of proteins can be described in terms of changes in particular solvent properties of aqueous media in their solutions, in addition to the size exclusion effect [12].

Crowding effects in polymer solutions are studied generally in order to simulate intracellular conditions existing *in vivo* [14,15,16,17]. These highly crowded conditions are achieved *in vivo* due to the overall concentration of a large variety of different proteins, nucleic acids, carbohydrates, and other solutes that may be as high as 400 g/L [14,15,16,17], while no individual macromolecular species are present there at such very high concentrations. As a result, biological macromolecules may occupy up to 40% of the cellular volume [14,15,16,17]. Although solvent properties of aqueous media under these conditions may be expected to be altered significantly, they have not been explored as of yet.

A solvatochromic analysis of solvent properties of aqueous media in protein solutions is hindered by the propensity of typical proteins to bind aromatic compounds, such as solvatochromic dyes. Therefore, as an attempt to examine the protein influence on solvent properties of aqueous media in their solutions, we have chosen an elastin-like polypeptide (ELP) composed of 40 pentapeptide (GVGVP) repeating units. The advantages of using this polypeptide in our study are: (a) it does not bind the solvatochromic dyes employed; and (b) ELPs are known to respond to changes in the composition of their environment in aqueous solutions by quantifiable changes in the readily measurable temperature of a phase transition, *T*_t_, also called the lower critical solution temperature (LCST) [18].

The purpose of this study was twofold—to quantify the solvent properties of aqueous ELP solutions in the presence of different salt additives known to affect the ELP phase transition temperature and in the presence of different osmolytes, and to explore if the phase transition temperature in these solutions might be described in terms of the solvent properties of the ELP solution. Additionally, it was necessary to verify that ELP does not bind the solvatochromic dyes used as probes of solvent properties in ELP solutions.

## 2. Results and Discussion

**Figure 1:**
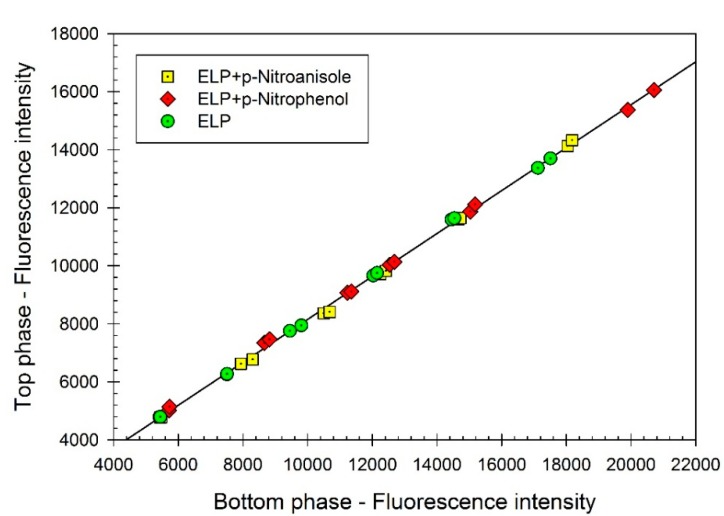
Concentration of elastin-like polypeptide (ELP) (represented by fluorescence intensity) in the top phase *vs.* concentration of ELP (represented by fluorescence intensity) in the bottom phase of aqueous Ficoll-PEG-0.01 M sodium phosphate buffer, pH 7.4 two-phase system. ELP, (GVGVP)_40_, was partitioned in the absence of additives (green circles), and in the presence of *p*-nitrophenol (red diamonds) or *p*-nitroanisole (yellow squares). Concentrations of ELP are measured in each phase with the *o*-phthaldialdehyde (OPA) assay. Slope of the linear curve represents the partition coefficient of ELP.

**Figure 2:**
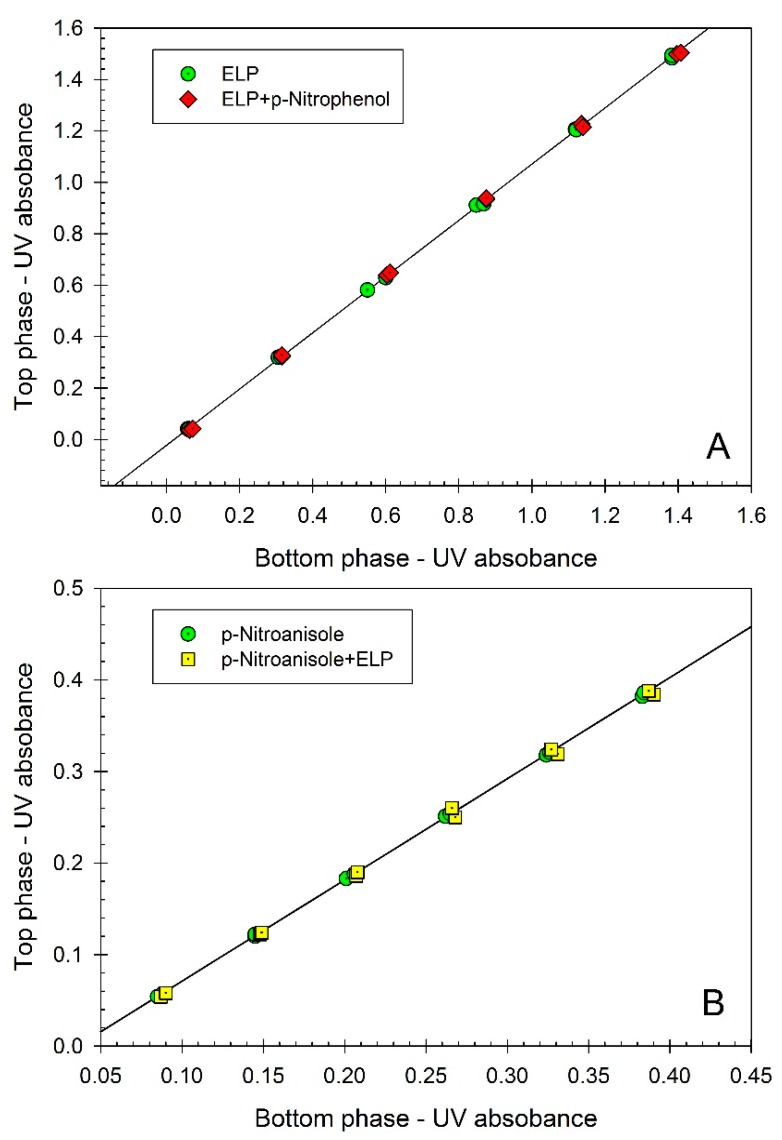

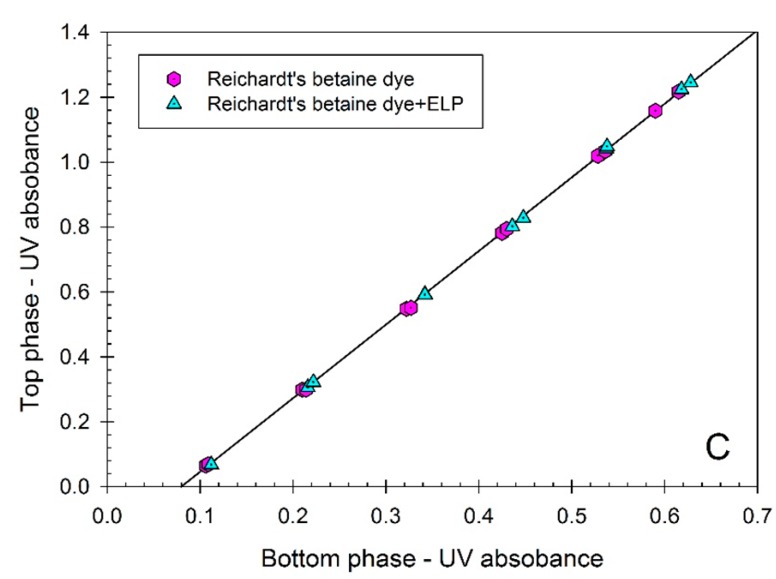
(**A**) Concentration of *p*-nitrophenol (represented by UV absorbance at 404 nm) in the top phase *vs.* concentration of *p*-nitrophenol (represented by UV absorbance at 404 nm) in the bottom phase of aqueous Ficoll-PEG-0.01 M sodium phosphate buffer, pH 7.4 two-phase system. *p*-Nitrophenol was partitioned in the absence of additives, and in the presence of ELP, (GVGVP)_40_. Concentrations of *p*-nitrophenol are measured in each phase following dilution with universal buffer, pH 12.4. Slope of the linear curve represents the partition coefficient of *p*-nitrophenol; (**B**) Concentration of *p*-nitroanisole (represented by UV absorbance at 318 nm) in the top phase *vs.* concentration of *p*-nitroanisole (represented by UV absorbance at 318 nm) in the bottom phase of aqueous Ficoll-PEG-0.01 M sodium phosphate buffer, pH 7.4 two-phase system. *p*-Nitroanisole was partitioned in the absence of additives, and in the presence of ELP, (GVGVP)_40_. Concentrations of *p*-nitroanisole are measured in each phase following dilution with water. Slope of the linear curve represents the partition coefficient of *p*-nitroanisole; (**C**) Concentration of the carboxylated Reichardt’s betaine dye (represented by UV absorbance at 308 nm) in the top phase *vs.* concentration of the carboxylated Reichardt’s betaine dye (represented by UV absorbance at 308 nm) in the bottom phase of aqueous Ficoll-PEG-0.01 M sodium phosphate buffer, pH 7.4 two-phase system. Carboxylated Reichardt’s betaine dye was partitioned in the absence of additives, and in the presence of ELP, (GVGVP)_40_. Concentrations of the carboxylated Reichardt’s betaine dye are measured in each phase following dilution with water. Slope of the linear curve represents the partition coefficient of the carboxylated Reichardt’s betaine dye.

In order to explore if the solvatochromic dyes bind to elastin-like polypeptide (ELP), we used the method of partitioning in aqueous two-phase system (ATPS). Partitioning of ELP in the Ficoll-70-PEG-8000-0.01 M sodium phosphate buffer, pH 7.4 was examined in the absence and in the presence of the two solvatochromic dyes (*p*-nitrophenol and *p*-nitroanisole) using *o*-phthaldialdehyde (OPA) fluorescence assay. The data presented graphically in Figure 1 show that the partition coefficient of ELP, *K*, remains the same (*K* = 0.740 ± 0.004) in the absence and in the presence of the dyes. It is well established that the partition coefficient of a given solute (an organic compound or a protein) in an aqueous two-phase system is extremely sensitive to binding of another compound [19,20,21]. Therefore, the data obtained by this approach may be viewed as confirming assumption that the ELP examined does not bind the dyes under study. In addition, we examined partitioning of all three dyes in the same ATPS in the absence and in the presence of ELP using UV/VIS absorbance assay. The results of these studies are shown in Figure 2. The partition coefficients of all three dyes: *p*-nitrophenol (*K* = 1.094 ± 0.003), *p*-nitroanisole (*K* = 1.103 ± 0.006), and Reichardt’s carboxylated betaine dye (*K* = 2.276 ± 0.005), do not change in the presence of ELP. These data confirm the notion that the dyes examined here do not interact with ELP and hence may be used as solvatochromic probes of the solvent properties of aqueous media in ELP solutions. Note that the partitioning of ELP in the presence of the Reichardt’s dye in the ATPS could not be examined because of the interference of the dye fluorescence with the OPA assay.

In order to estimate the highest ELP concentration that could be used for solvatochromic measurements, we first explored the UV/Vis spectra of one of the solvatochromic dyes—4-nitroanisole—in solutions of ELP in an aqueous 0.01 M sodium phosphate buffer (NaPB) at pH 7.4 over the ELP concentration range of 5 to 25 mg/mL. ELP solutions display a rather high intensity absorption maximum at λ ≈ 280 nm due to the presence of a tryptophan residue close to the N-terminus of the polypeptide chain.

The tail of the absorption peak at 280 nm was found to mask the UV absorption maximum of 4-nitroanisole at ≈317 nm at high ELP concentrations. The highest ELP concentration at which the solvatochromic shift of the dye absorption maximum could be determined reliably was found to be 15 mg/mL (0.90 mM) ELP.

**Figure 3:**
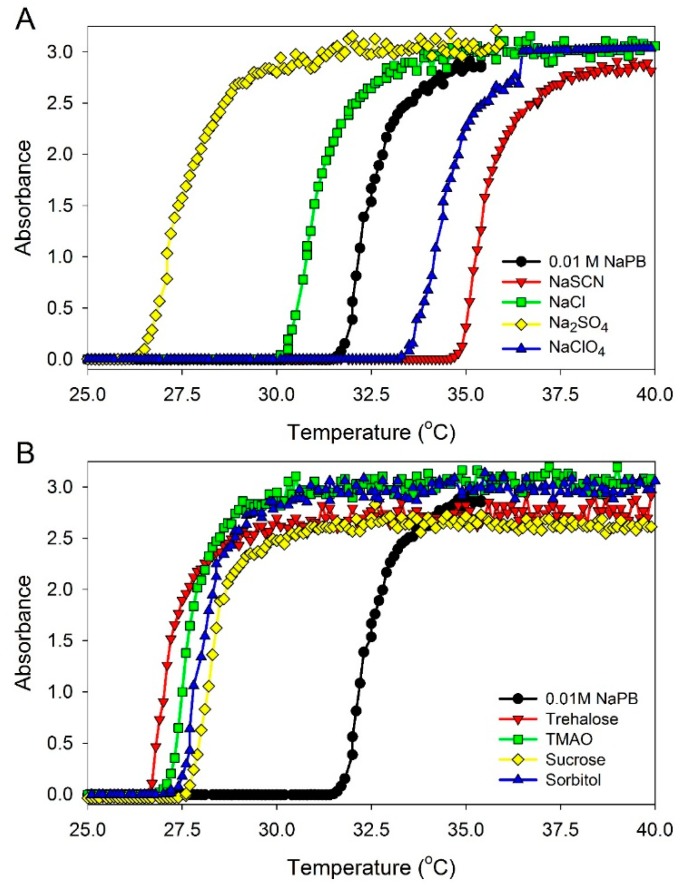
UV absorbance at 350 nm of (GVGVP)_40_ at 0.90 mM concentration in 0.01 M sodium phosphate buffer (NaPB), pH 7.4 in the presence of different additives as a function of temperature. Additives: (**A**) salts—0.1 M NaCl, 0.05 M Na_2_SO_4_, 0.1 M·NaClO_4_, and 0.1 M NaSCN; and (**B**) osmolytes—0.5 M sucrose, 0.4 M sorbitol, 0.4 M trehalose, and 0.4 M trimethylamine *N*-oxide (TMAO). In order to determine the transition temperature for ELP solutions, the samples were heated at a rate of 1 °C/min and the UV absorbance at 350 nm was recorded.

The phase-transition temperatures in ELP solutions at a 0.90 mM concentration in the presence of different additives were measured as described in Materials and Methods (see below). The data obtained are illustrated in Figure 3 and are listed in Table 1. It should be mentioned that all solvatochromic measurements were performed at 23 °C, and the concentrations of the additives were adjusted for this temperature to be below the phase transition temperature, *T*_t_, in ELP solutions at the ELP concentration of 0.90 mM. It should also be noted that the addition of osmolytes known to stabilize the protein structure in solution [22,23,24] were found to decrease the phase transition temperature of ELP. Table 1 also shows that the addition of ELP moderately affected hydrogen bond donor acidity (α) and dipolarity/polarizability (π*) of solutions containing different salts and osmolytes, whereas hydrogen bond acceptor basicity (β) of the same solutions of small additives (except to trimethylamine *N*-oxide (TMAO)) was affected to a much lesser degree.

**Table 1:** Solvent properties π*, α, and β of aqueous media in 10 mM sodium phosphate buffer at pH 7.4 in the presence of different additives and phase-transition temperature, *T*_t_, as well as solvent properties in solutions of 0.90 mM ELP in the presence of additives *.

Additive	*T*_t_/°C	Solvent Properties of Aqueous Media		
		Dipolarity/Polarizability, π*	H-Bond Acidity, α	H-Bond Basicity, β
0.01 M NaPB	31.6 ± 0.1	1.118 ± 0.001	1.186 ± 0.001	0.594 ± 0.003
0.01 M NaPB		1.104 ± 0.002	1.248 ± 0.002	0.596 ± 0.002
0.1 M NaCl	30.2 ± 0.1	1.115 ± 0.003	1.198 ± 0.003	0.602 ± 0.006
0.1 M NaCl		1.111 ± 0.001	1.248 ± 0.001	0.597 ± 0.001
0.05 M Na_2_SO_4_	26.5 ± 0.1	1.114 ± 0.002	1.183 ± 0.002	0.611 ± 0.007
0.05 M Na_2_SO_4_		1.107 ± 0.002	1.248 ± 0.002	0.603 ± 0.001
0.1 M NaClO_4_	33.3 ± 0.1	1.120 ± 0.003	1.192 ± 0.002	0.605 ± 0.005
0.1 M NaClO_4_		1.114 ± 0.001	1.257 ± 0.001	0.597 ± 0.001
0.1 M NaSCN	34.9 ± 0.1	1.124 ± 0.004	1.180 ± 0.003	0.604 ± 0.005
0.1 M NaSCN		1.118 ± 0.001	1.238 ± 0.002	0.597 ± 0.001
0.5 M sucrose	27.6 ± 0.1	1.140 ± 0.003	1.143 ± 0.003	0.603 ± 0.002
0.5 M sucrose		1.136 ± 0.002	1.192 ± 0.003	0.606 ± 0.003
0.4 M sorbitol	27.4 ± 0.1	1.129 ± 0.007	1.174 ± 0.005	0.591 ± 0.006
0.4 M sorbitol		1.120 ± 0.002	1.241 ± 0.007	0.601 ± 0.003
0.4 M trehalose	26.2 ± 0.1	1.141 ± 0.003	1.154 ± 0.003	0.608 ± 0.005
0.4 M trehalose		1.127 ± 0.002	1.204 ± 0.002	0.606 ± 0.003
0.4 M TMAO	27.2 ± 0.1	1.115 ± 0.004	1.187 ± 0.003	0.623 ± 0.003
0.4 M TMAO		1.099 ± 0.001	1.233 ± 0.003	0.638 ± 0.002

* The solvent properties of ELP solutions are in the rows with the *T*_t_ indicated.

**Figure 4:**
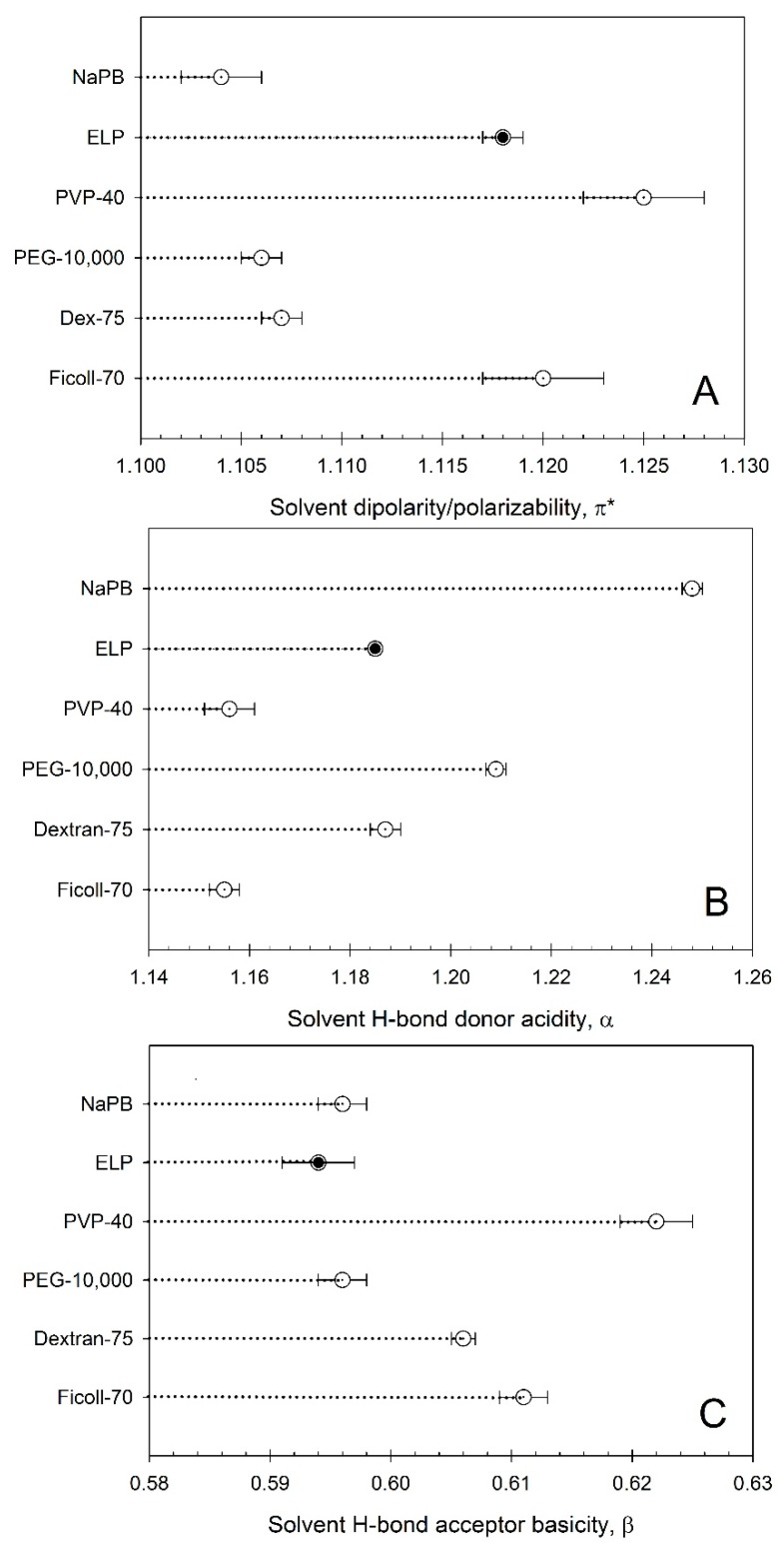
Solvent properties of aqueous media in solutions of ELP (GVGVP)_40_ and nonionic polymers at a 0.90 mM concentration in an aqueous 0.01 M sodium phosphate buffer (NaPB) pH 7.4: (**A**) dipolarity/polarizability π*; (**B**) H-bond donor acidity α; and (**C**) H-bond acceptor basicity β. Ficoll-70—Ficoll with a weight-averaged molecular mass of *M*_W_ ~ 70,000; dextran-75—dextran with *M*_W_ ~ 75,000; PEG-10,000—polyethylene glycol with *M*_W_ ~ 10,000; PVP-40—polyvinylpyrrolidone with *M*_W_ ~ 40,000; and NaPB—0.01 M sodium phosphate buffer, pH 7.4.

The solvent features of aqueous media for 0.90 mM ELP in 0.01 M NaPB are plotted in Figure 4A–C. This data is compared to previously reported data [12] for different non-ionic polymers commonly used as crowding agents at the same concentration.

It can be seen that the solvent’s dipolarity/polarizability values π* (Figure 4A), characterizing the solvent’s ability to be involved in dipole and dipole/induced dipole interactions with a solute is within the range observed for non-ionic polymers—between those observed for Ficoll-70 and Dextran-75 at the same concentration. The solvent’s HBD acidity values, α, characterizing the solvent’s ability to participate as donor in hydrogen bonding with a solute, is also within the range observed for nonionic polymers—between those observed for Ficoll-70 and Dextran-75 (Figure 4B). The solvent’s HBA basicity values, β, of aqueous media in the ELP solution, characterizing the solvent’s ability to participate as acceptor in hydrogen bonding with a solute, is very small—close to that observed for PEG-10,000 (Figure 4C) [12].

An intricate feature of ELPs (which are repeats of the GVGXaaP pentapeptide), is the ability of these polypeptides to undergo a reversible phase transition from the intrinsically disordered, highly solvated conformation below the inverse transition temperature (*T*_t_) to a new phase comprised of a dehydrated aggregated polypeptide (referred to as a coacervate) when the temperature is raised above *T*_t_ [25,26,27,28]. It is well known that the *T*_t_ value of an ELP is a concentration-dependent parameter that is inversely related to its concentration, the number of monomer repeats, and the hydrophobicity of the variable Xaa residue of the pentapeptide repeat [26,29,30,31].

The data obtained here may explain the results reported by Ge *at al*. [32], according to which, the expression of ELP or ELP-GFP fusion protein in *E. coli* resulted in the formation of an aqueous two-phase system (ATPS) in the cytoplasm of the cell.

One of the phases appeared to serve as depot for newly formed protein and excluded the cellular proteases [32]. The formation of such intracellular ATPS may be due to the effects of ELP on the solvent properties of cytoplasmic aqueous media. It should be mentioned, however, that the ELP described in [32] consisted of 90 repeats of the VPGXaaG pentapeptide, where Xaa was V, A, and G in the ratio 5:2:3. Therefore, the effects of this ELP on the solvent properties of water may differ from those determined in our study.

Besides the aforementioned polypeptide structure related parameters, the *T*_t_ of an ELP can be modulated by changes in the solvent composition. It varies in the presence of different co-solutes in accordance with their stabilizing/destabilizing effects on the temperature-induced coacervate phase of an ELP [18]. Therefore, the aforementioned co-solute-induced decrease/increase in the *T*_t_ is a reflection of the increased/decreased conformational stability of the coacervate in the presence of a given additive.

In line with these considerations, the phase-transition temperature *T*_t_ for the ELP solution (the ELP used in our study is MGH-(GVGVP)_40_-GWP in the presence of different additives (see Table 1)) changes in the following sequence: 0.4 M trehalose < 0.05 M Na_2_SO_4_ < 0.4 M TMAO ≤ 0.4 M sorbitol ≤ 0.5 M sucrose < 0.1 M NaCl < 0.01 M NaPB < 0.1 M NaClO_4_ < 0.1 M NaSCN; *i.e.*, it decreases relative to the *T*_t_ for the buffer without additives in the presence of osmolytes, known to stabilize the polypeptide structure, and in the presence of sulfate and chloride; it increases in the presence of perchlorate and thiocyanate known to destabilize the polypeptide structure, in agreement with the data reported by Cremer *et al.* [18,33]. It should be reemphasized here that the different concentrations of salts and osmolytes additives were used in our study because all the solvatochromic measurements of the solvent properties of aqueous media in ELP solutions were performed at 23 °C, and each additive concentration had to be adjusted for the ELP solution to be below the phase-transition temperature *T*_t_.

It has been found previously that the yield of protein refolding in the presence of crowding polymers may be described in terms of the dipolarity/polarizability, π*, and HBD acidity, α, of aqueous media [12]. Therefore, we explored similar relationships for phase-transition temperature of ELP in the presence of different additives. The HBA basicity, β, is very lightly affected in polymer solutions as well as in ELP solutions, and hence appears not to be an important factor [12].

**Figure 5:**
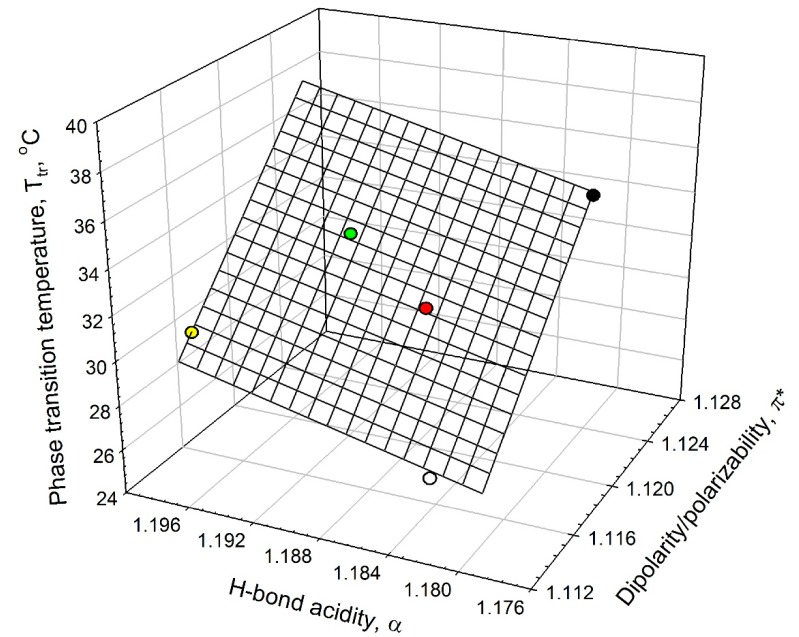
Interrelationship between the phase-transition temperature, *T*_t_, in ELP solutions and the solvent dipolarity/polarizability, π*, and H-bond donor acidity, α, in ELP solutions at 0.90 mM concentration in an aqueous 0.01 M sodium phosphate buffer (NaPB) of pH 7.4 (black circle) in the presence of 0.1 M·NaCl (white circle), 0.05 M Na_2_SO_4_ (red circle), 0.1 M NaClO_4_ (green circle), and 0.1 M·NaSCN (yellow circle) (see Equation (2)).

Analysis of the data listed in Table 1 shows that the ELP phase-transition temperature, *T*_t_, in salt solutions is linearly related to the solvent’s dipolarity/polarizability, π*, and HBD acidity, α, as shown graphically in Figure 5. This linear relationship can be described as in Equation (2):

*T*_t_ = −(1146 ± 133) + (871 ± 88) π* + (167.50 ± 49) α; *N* = 5; *r*^2^ = 0.9804; SD = 0.6; *F* = 50 (2)

where π* and α parameters are the solvent’s dipolarity/polarizability and HBD acidity in the aqueous solutions of 0.90 mM ELP in the presence of the additives; *N* is the number of additives (NaPB, Na_2_SO_4_, NaCl, NaClO_4_, and NaSCN), *r* is the correlation coefficient; SD is the standard deviation; and *F* is the ratio of variance. It should be emphasized that the number of the experimental data used in relationship described by Equation (2) is extremely small (*N* = 5), and hence the aforementioned relationship cannot be viewed as sufficiently solid and should be considered as a trend and not as a reliable correlation.

Further analysis shows that the ELP phase-transition temperature in the presence of non-ionic osmolytes additives can also be described in terms of the same solvent properties as:

*T*_t_ = (384.9 ± 20) − (202.9 ± 11) π* − (110.2 ± 6) α; *N* = 4; *r*^2^ = 0.9971; SD = 0.1; *F* = 171.2 (3)

where *N* is the number of additives (sucrose, sorbitol, TMAO, or trehalose), and all the other parameters are as defined before. It should be stressed that given the small number of experimental data and very limited range of the *T*_t_ values, the relationship described by Equation (3) should be viewed as a trend and not as a reliable correlation. At the moment, it remains unclear why the data for ELP phase transition temperature in 0.01 M phosphate buffer do not fit Equation (3). We are planning to perform additional experiments to better understand mechanisms defining correlations between the ELP phase transition temperatures and solvent properties of ELP solutions in the presence of different additives and to find the factors affecting the reliability and applicability of linear equations for real situations.

It follows from comparison of the relationships described by Equations (2) and (3) that the solvent’s dipolarity/polarizability is the primary solvent property affecting the phase-transition temperature in 0.90 mM ELP solutions in the presence of both salts and nonionic osmolytes additives.

The quantitative differences between the two Equations (2) and (3) are likely due to the different mechanisms of the influence of salts and osmolytes on the ELP phase-transition temperature. According to Zhang and Cremer [34], these mechanisms may be divided into two categories: direct and indirect effects.

The direct mechanisms are defined by Zhang and Cremer [34] as those involving hydrogen bonding between co-solute molecules/ions and polypeptide backbone and/or polar and charged side-chains.

Indirect effects are defined as influences of co-solute molecules/ions on the solvation of polypeptide or changes to bulk water properties [34]. The influence of salt additives are generally viewed as direct [34], whereas the effects of osmolytes, on the other hand, may be indirect [34] and are likely realized as changes in the polypeptide solvation via the influence of osmolytes on the interfacial or bulk water properties.

The main conclusion from both Relationships (1) and (2) is that the temperature of phase transitions in the ELP solutions in the presence of different additives is interrelated with the solvent properties of aqueous media in the ELP solutions.

The results obtained in this study clearly raise multiple questions that cannot be answered yet. The most important questions are—is the change in the ELP phase-transition temperature caused by changes in the solvent properties of aqueous media, and what are the molecular mechanisms of the ELP-induced changes in the solvent properties of aqueous media? The important conclusion is that the solvatochromic approach used here is applicable to studying polypeptides and may be feasible for studying some proteins as well. Further studies are clearly needed to answer these questions and they are currently in progress in our laboratories.

## 3. Experimental Section

### 3.1. Elastin-Like Polypeptide (ELP) Gene Design

The gene encoding of the ELP was produced as previously reported [35,36] using a modification of the recursive directional ligation methods [37]. Complementary oligonucleotides (Invitrogen, Thermo Fisher Scientific Inc., Waltham, MA, USA) encoding five repeats of the amino acid sequence GVGVP were designed to have appropriate overhangs to insert into a pUC19 cloning vector (Novagen, EMD Millipore, Billerica, MA, USA). Two separate aliquots of the pUC19 containing ELP were then double digested using restriction enzymes (NdeI, BglI and PflmI), and ligated together in order to increase the length of the ELP. The process was repeated 3 times to make 40 repeats. The (GVGVP)_40_ encoding DNA was then ligated into a pET20b expression vector and transformed using a BL21* (DE3) strain of *E. coli*. The resulting gene encodes a polypeptide of 206 amino acids: MGH(GVGVP)_40_-GWP, which was verified by DNA sequencing (Cleveland Clinic, Cleveland, OH, USA).

### 3.2. Protein Expression, Purification, and Characterization

To express the protein, two 10 mL starter cultures from a frozen stock were prepared in a LB medium supplemented with 100 μg/mL ampicillin at 37 °C. The starter culture was added to 1 L of the LB medium, shaken at 300 rpm and 37 °C to an OD_600_ of 1.0, at which point the expression was induced by adding 0.1 mM IPTG (Fisher Scientific). The cells were harvested after 4 to 5 h by centrifugation for 30 min at 7000× *g*. The pellet was re-suspended in filtered H_2_O and lysed by a sonic dismembrator. The lysed cells were then centrifuged at 4 °C for 20 min at 20,000× *g* to separate the soluble protein from the insoluble fraction. The soluble supernatant, which contains the protein, was then purified using the inverse transition-cycling method [38]. The protein was heated to 55 °C for at least 4 h and then centrifuged for 15 min at about 40 °C at 20,000× *g* to pellet out the insoluble portion. The isolated pellet was re-suspended in 5 mL of filtered H_2_O and centrifuged at 4 °C for 15 min at 20,000× *g*. This process was repeated two times to purify the ELP. Protein purity and molecular mass confirmation was performed using SDS-PAGE with 4%–20% gradient Tris-HEPES-SDS gel (Pierce, Rockford, IL, USA). Samples were prepared in loading buffer containing 1% SDS. Molar concentrations of the purified proteins were determined based on calculated extinction coefficients and absorbance at λ = 280 nm, measured on a Biomate 3 spectrophotometer (Thermo Scientific, Rockford, IL, USA).

### 3.3. Transition-Temperature Measurements

Purified ELP was lyophilized and solutions of 15 mg/mL concentration were made in filtered H_2_O. The transition temperature (*T*_t_) of the protein was determined by measuring the UV-absorbance of solutions at λ = 350 nm on a Shimadzu 1800 UV–Vis spectrophotometer with an attached temperature control cell. Each solution contains 0.01 M NaPB of pH 7.4 added to it, followed by addition of appropriate amounts of either salts or osmolytes, added gravimetrically to a 2 mL sample in a quartz cuvette. The *T*_t_ measurements were stepwise run from 20 to 50 °C using a step of 0.1 °C and a temperature ramp of 1 °C/ min. The data were plotted as temperature *vs.* absorbance. The *T*_t_ is measured as the onset of turbidity determined from the intersection of the tangent lines of zero absorbance and the highest slope of the curve on a UV absorbance spectrum [39].

### 3.4. Solvatochromic Measurements

#### 3.4.1. Solvatochromic Dyes

**Figure 6:**
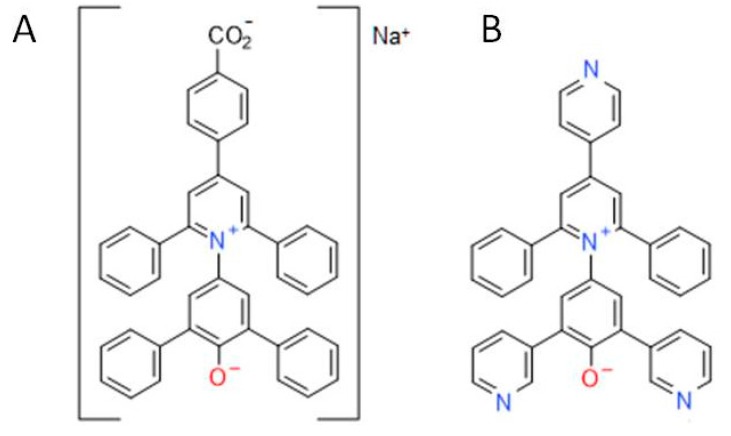
Molecular structure of two Reichardt’s dyes used in this study. (**A**) Carboxylated betaine dye used in partition experiments; (**B**) 4-[2,6-Diphenyl-4-(pyridine-4-yl)pyridinium-1-yl]-2,6-bis(pyridine-3-yl)phenolate, also known as Reichardt’s pyridyl-substituted *E*_T_(8) betaine dye [40], used as a solvatochromic probe.

The solvatochromic probe 4-nitrophenol (spectrophotometric grade) was purchased from Sigma (St. Louis, MO, USA). The 4-nitroanisole probe (GC, >99%) was supplied by Acros Organic (New Jersey, NJ, USA). 4-[2,6-Diphenyl-4-(pyridine-4-yl)pyridinium-1-yl]-2,6-bis(pyridine-3-yl)phenolate (Reichardt’s pyridyl-substituted E_T_(8) betaine dye) was synthesized as described in [40,41]. Carboxylated form of this betaine dye was used in partition experiments. The molecular structures of both dyes are shown in Figure 6.

#### 3.4.2. Other Chemicals

All salts and other chemicals used were of analytical-reagent grade. Deionized water was used for the preparation of all solutions.

#### 3.4.3. Solvatochromic Studies

All ELP solutions were prepared in a 0.01 M sodium phosphate buffer of pH 7.4 (NaPB) with and without salts and osmolytes additives by weight. The solvatochromic probes, 4-nitroanisole, 4-nitrophenol, and Reichardt’s E_T_(8) betaine dye, were used to determine the dipolarity/polarizability π*, H-bond acceptor (HBA) basicity β, and H-bond donor (HBD) acidity, α, of the aqueous media in which the ELP polymer was dissolved.

Aqueous solutions (~10 mM) of each solvatochromic dye were prepared and from 4 to 48 μL of the dye solution was added to the ELP solution to a total volume of 210 μL. A strong base was added to the samples (~2 μL 1 M NaOH) containing Reichardt’s E_T_(8) betaine dye, to ensure a basic pH. A strong acid (~4 μL 1 M HCl) was added to the samples containing 4-nitrophenol in order to eliminate charge-transfer bands of the phenolate anion. The respective blank solutions without any dye were prepared separately. The samples were mixed thoroughly in a vortex mixer and the absorption spectra of each solution were acquired. To estimate the reproducibility, possible aggregation, and specific interactions effects, the position of the band maximum in each solution was measured in three separate aliquots of each solution. A UV–Vis microplate reader spectrophotometer SpectraMax Plus384 (Molecular Devices, Sunnyvale, CA, USA) with a bandwidth of 2.0 nm, a data interval of 1 nm, and a high resolution scan (~0.5 nm/s) was used for acquisition of the UV–Vis molecular absorbance data. The absorption spectra of each probe were determined over the spectral range from 240 to 600 nm in each ELP solution in 0.01 M sodium phosphate buffer with and without additives. Pure solutions containing no dye (blank) were scanned first to establish the baseline. The wavelength of maximum absorbance was determined as described by Huddleston *et al.* [42]. The maximum wavelength was determined using PeakFit software package (Systat Software Inc., San Jose, CA, USA) as the average between the results obtained in all these scans. Average standard deviations for each measured wavelength were ≤0.4 nm for all probes. The results of the solvatochromic studies were used to calculate π*, β, and α as described by Marcus [43].

#### 3.4.4. Determination of the Solvent Dipolarity/Polarizability π*

π* values were determined from the wavenumber (v_1_) of the longest-wavelength Vis absorption band of 4-nitroanisole using Equation (4) [43]:

π* = 0.427(34.12 − v_1_) (4)

#### 3.4.5. Determination of the Solvent Hydrogen-Bond Acceptor (HBA) Basicity β

Each β value was determined from the wavenumber (v_2_) of the longest-wavelength Vis absorption band of 4-nitrophenol using Equation (5) [43]:

β = 0.346(35.045 − v_2_) − 0.57 π* (5)

#### 3.4.6. Determination of the Solvent Hydrogen-Bond Donor (HBD) Acidity α

The α values were determined from the longest-wavelength Vis absorption band of the 4-[2,6-diphenyl-4-(pyridine-4-yl)pyridinium-1-yl]-2,6-bis(pyridine-3-yl)phenolate (Reichardt’s E_T_(8) betaine dye) using the Equations (6) to (8):

E_T_(8)/(kcal·mol^−1^) = 28,591/λ_max_ (nm) (6)

In Equation (6), λ_max_ is the wavelength of the maximum of the long-wavelength solvatochromic absorption band of betaine dye 8.

The empirical Reichardt’s solvent polarity index, E_T_(30), was then calculated from the E_T_(8) values with the following linear relationship for HBD solvents [40]:

E_T_(30) = [E_T_(8) − 16.236]/0.704 (7)

Finally, the α values were calculated from the E_T_(30) values according to Equation (8) [43]:

α = 0.0649·E_T_(30) − 2.03 − 0.72 π* (8)

#### 3.4.7. Partitioning of ELP and Solvatochromic Dyes in the Aqueous PEG-8000-Ficoll-70 Two-Phase System

Ficoll-70 (lot 128K1136), with weight-average molecular weight (*M*_W_) ~ 70,000) and polyethylene glycol PEG-8000 (Lot 091M01372V) with an average molecular weight (*M*_n_) of 8000 were purchased from Sigma-Aldrich. Reichardt’s carboxylated betaine dye (2,6-diphenyl-4-[4-(4-carboxylatophenyl)-2,6-diphenylpyridinium-1-yl])phenolate) was synthesized according to the procedure reported previously [44] and used in the partitioning experiments because this dye was at our disposal in larger quantities than the Reichardt’s dye (8) used in solvatochromic measurements and it has the structure essentially similar to that of the Reichardt’s dye (8) (see Figure 6).

Stock solutions of PEG 8000 (50% wt.) and Ficoll-70 (~49% wt.) were prepared in deionized (DI) water. Stock sodium phosphate buffer (NaPB; 0.5 M, pH 7.4) was prepared by mixing appropriate amounts of NaH_2_PO_4_ and Na_2_HPO_4_. A mixture of polymers was prepared by dispensing appropriate amounts of the aqueous stock polymer solutions into a 1.2 mL microtube using a Hamilton Company (Reno, NV, USA) ML-4000 four-probe liquid-handling workstation. Appropriate amounts of stock buffer solutions and water were added to give the ionic and polymer composition required for the final system (after the sample addition—see below) with total weight of 0.5 g (total volume 462 ± 1 μL). All the aqueous two-phase systems used had the same polymer composition of 9.0% wt. PEG-8000 and 19.0% wt. Ficoll-70 and same ionic composition of 0.01 M NaPB, pH 7.4.

An automated instrument for performing aqueous two-phase partitioning, the Automated Signature Workstation, ASW (Analiza, Inc., Cleveland, OH, USA), was used for the partitioning experiments. The ASW system is based on the ML-4000 liquid-handling workstation (Hamilton Company) integrated with a FL600 fluorescence microplate reader (Bio-Tek Instruments, Winooski, VT, USA) and a UV–Vis microplate spectrophotometer (SpectraMax Plus 384, Molecular Devices, Sunnyvale, CA, USA). Solutions of ELP were prepared in water at concentration of 5 mg/mL. The ELP solution was also mixed with 10 mM solution of p-nitrophenol in the 3:1 ratio by volume and with 3 mM *p*-nitroanisole solutions in the 1:1 ratio by volume. Varied amounts (0, 15, 30, 45, 60 and 75 μL) of ELP solution or ELP-dye mixture and the corresponding amounts (75, 60, 45, 30, 15 and 0 μL) of water were added to a set of the same polymers/buffer mixtures. The systems were then vortexed in a Multipulse vortexer and centrifuged (Jouan, BR4i, Thermo Fisher Scientific, Waltham, MA, USA) for 60 min at 3500× *g* at 23 °C to accelerate phase settling. The top phase in each system was removed, the interface discarded, and aliquots from the top and bottom phases were withdrawn in duplicate for analysis by OPA assay (see below).

Solutions of *p*-nitrophenol and *p*-nitroanisole at the concentrations of 10 and 3 mM, respectively, and solution of carboxylated betaine Reichardt’s dye at concentration of 4.2 mM were prepared in water. These solutions and their mixtures with ELP solution in the 1:3 ratio (for *p*-nitrophenol) and 1:1 ratio (for two other dyes) by volume were prepared. All these solutions of individual dyes and their mixtures with ELP were used for partitioning as described above, except that the aliquots from the top and bottom phases were withdrawn in duplicate for analysis by UV/Vis assay (see below).

For the analysis of ELP partitioning, aliquots of 30 µL from both phases were transferred and diluted with water up to 70 µL into microplate wells. Then, the microplate was sealed, shortly centrifuged (2 min at 1500 rpm) and following moderate shaking for 45 min in an incubator at 37 °C, 250 µL of *o*-phthaldialdehyde reagent was added. After moderate shaking for 4 min at room temperature, fluorescence was determined using a fluorescence plate reader with a 360 nm excitation filter and a 460 nm emission filter, with a sensitivity setting of 100–125.

For the analysis of the dyes partitioning in the presence or absence of ELP, 50–120 µL aliquots from both phases were diluted up to 600 µL in 1.2 mL microtubes. Water was used as diluent except *p*-nitrophenol, in the case of which 20 mM universal buffer with pH 12.4 was used as diluent. Universal buffer is composed of 0.01 M each of phosphoric, boric, and acetic acids adjusted to pH 12.4 with NaOH. Following vortexing and a short centrifugation (12 min), aliquots of 250–300 µL were transferred into microplate wells, and the UV–Vis plate reader was used to measure optical absorbance at wavelengths previously determined to correspond to maximum absorption. The maximum absorption wavelength for each compound was determined in separate experiments by analysis of the absorption spectrum over the 240–500 nm range. In the case of *p*-nitrophenol, the maximum absorption was found to be more concentration sensitive in the presence of the universal buffer at pH 12.4. In all measurements, the same dilution factor was used for the upper and lower phases and correspondingly diluted pure phases were used as blank solutions.

The partition coefficient, *K*, is defined as the ratio of the sample concentration in the top phase to that in the bottom phase. The *K-*value for each solute was determined as the slope of the concentration (fluorescence intensity or absorbance depending on the compound) in the top phase plotted as a function of the concentration in the bottom phase averaged over the results obtained from two to four partition experiments carried out at the specified composition of the system (see Figure 2 and Figure 3).

## 4. Conclusions

In conclusion, we investigated the effect of elastin-like polypeptide on solvent properties of aqueous media. To this end, several solvent properties in aqueous solutions of an elastin-like polypeptide (ELP) with the (GVGVP)_40_ sequence were quantified using the so-called solvatochromic comparison method. The studied solvent properties included solvent dipolarity/polarizability, hydrogen-bond donor acidity, and hydrogen-bond acceptor basicity. The phase-transition temperatures under the variety of conditions were investigated as well. The phase-transition temperatures and the solvent properties were examined in solutions of 0.90 mM ELP in 0.01 M sodium phosphate buffer, at pH 7.4, in the presence of different salt additives (Na_2_SO_4_, NaCl, NaClO_4_, and NaSCN) and various osmolyte additives (sucrose, sorbitol, trehalose, and TMAO). We showed that all osmolyte additives at 0.4–0.5 M decreased the phase-transition temperature of ELP, while Na_2_SO_4_ and NaCl (0.05 and 0.1 M) decreased it, and NaClO_4_ and NaSCN (0.1 M) increased it. The determined phase-transition temperatures may be described as a linear combination of the solvent’s dipolarity/polarizability and hydrogen-bond donor acidity. The linear relationship observed for the phase-transition temperature in the presence of salt additives differs quantitatively from that in the presence of osmolyte additives, in agreement with different (direct and indirect) mechanisms of the influence of salts and osmolytes on the ELP phase-transition temperature.
